# The role of *HoxA11* and *HoxA13* in the evolution of novel fin morphologies in a representative batoid (*Leucoraja erinacea*)

**DOI:** 10.1186/s13227-017-0088-4

**Published:** 2017-12-01

**Authors:** Shannon N. Barry, Karen D. Crow

**Affiliations:** Department of Biology, San Francisco State University, 1600 Holloway Ave, San Francisco, CA 94127 USA

**Keywords:** HoxA, Skate, Fin, AER, Clasper, Hox

## Abstract

**Background:**

Batoids exhibit unique body plans with derived fin morphologies, such as the anteriorly expanded pectoral fins that fuse to the head, or distally extended anterior pelvic fin lobes used for a modified swimming technique utilized by skates (Rajidae). The little skate (*Leucoraja erinacea*), exhibits both of these unique fin morphologies. These fin modifications are not present in a typical shark body plan, and little is known regarding the mechanisms underlying their development. A recent study identified a novel apical ectodermal ridge (AER) associated with the development of the anterior pectoral fin in the little skate, but the role of the posterior HoxA genes was not featured during skate fin development.

**Results:**

We present the first evidence for HoxA expression (*HoxA11* and *HoxA13*) in novel AER domains associated with the development of three novel fin morphologies in a representative batoid, *L. erinacea*. We found *HoxA13* expression associated with the recently described novel AER in the anterior pectoral fin, and *HoxA11* expression in a novel AER domain in the anterior pelvic fin that we describe here. We find that both *HoxA11* and *HoxA13* are expressed in claspers, and while *HoxA11* is expressed in pelvic fins and claspers, *HoxA13* is expressed exclusively in developing claspers of males. Finally, *HoxA11* expression is associated with the developing fin rays in paired fins.

**Conclusion:**

Overall, these results indicate that the posterior HoxA genes play an important role in the morphological evolution of paired fins in a representative batoid. These data suggest that the batoids utilize a unique Hox code, where the posterior HoxA genes exhibit distinct expression patterns that are likely associated with specification of novel fin morphologies.

**Electronic supplementary material:**

The online version of this article (10.1186/s13227-017-0088-4) contains supplementary material, which is available to authorized users.

## Background

Vertebrates exhibit an impressive array of morphological diversity, with a variety of body plan features exhibited by extinct and extant taxa, and the cartilaginous fishes are no exception. Cartilaginous fishes represent the most ancestral lineage of extant jawed vertebrates, which diverged from the bony fishes approximately 420–450 million years ago [1, 2], and include the holocephalans, sharks, rays and skates. Therefore, cartilaginous fishes provide an important phylogenetic context for comparing fin and limb development in gnathostomes, and understanding the genetic underpinnings for trait variation and body plan diversity [3]. Approximately half of all cartilaginous fishes exhibit a modified body plan that is dorsoventrally flattened, with large pectoral fins that expand anteriorly and fuse to the head or rostrum (Batoidea). A compelling focus of evolutionary developmental studies has been the evolution of modified appendages such as the fin to limb transition, and these studies have highlighted co-option of ancestral genetic regulatory networks (GRNs) that were present in the common ancestor of jawed vertebrates as a primary mechanism in the evolution of body plan disparity [3, 4], including novel deployments of HoxA/D patterning genes.

Hox genes are classically associated with early patterning of anterior and posterior domains during embryogenesis and have the intriguing property of collinear expression, in which the genes on the 3′ end of the cluster are expressed early in anterior domains, followed by expression of the 5′ genes in more restricted and posterior domains. Later in development, HoxA/D genes play an important role during fin and limb morphogenesis, and are commonly referred to as the “fin and limb building toolkit” [5]. The expression patterns of the posterior HoxD genes have been well characterized and are associated with specification of morphological disparity via anterioposterior patterning of distal domains. The 5′ HoxA genes specify proximal/distal domains and also have been implicated in the morphogenesis of novel features in distal domains [5, 6]. In tetrapods, the posterior HoxA genes, *HoxA11* and *HoxA13,* have mutually exclusive expression patterns with *HoxA11* patterning the forearm (zeugopod), and *HoxA13* patterning the hand (autopod) [7–9]. *HoxA11* and *HoxA13* expression is overlapping during pectoral fin development in shark [10], paddlefish [11, 12] and zebrafish [13], suggesting that exclusive expression domains in the autopod is a tetrapod novelty [14].

Fins and limbs develop as outgrowths of the body wall and are patterned by two signaling centers referred to as the apical ectodermal ridge (AER), which is associated with distal outgrowth, and the zone of polarizing activity (ZPA) which is associated with AP patterning [15]. The AER plays a major role in the patterning and outgrowth of fins and limbs, and removal of the AER results in the loss of distal elements in the limbs of tetrapods [16]. AER outgrowth and patterning is accomplished via interactions with major signaling pathways involving *Wnt3* and *fibroblast growth factor 10* (*Fgf10*) [16, 17]. During fin development in ray-finned fishes, the AER flattens into an apical fold (AF) that will eventually give rise to the dermal fin rays [18]. However, in tetrapods, development of the AF is delayed, resulting in continued AER signaling and distal elongation of endoskeletal elements including the eventual development of digits [19]. *Wnt3* is an established AER marker and is associated with underlying *Fgf* signaling, along with bone morphogenetic proteins (BMP) and *Notch1* [20, 21]. These genes maintain the AER within the developing fin and limb mesenchyme [17, 22]. For example, during early pectoral fin development of the little skate, the distal AER is maintained by the presence of *Wnt3* and *Fgf10*, along with expression of the 5′ HoxD genes [20, 23].

The establishment of novel AER domains is associated with the evolution of novel fin/limb morphologies. In addition to the extended AER domain promoting endoskeletal outgrowth in tetrapods, a novel AER domain was recently described in the anterior pectoral fin of the little skate that is associated with a novel morphology including anterior expansion and fusion to the pharyngeal basket, head, and rostrum [20]. Nakamura et al. [20] demonstrated that there is an additional and distinct AER in the anterior region of the little skate pectoral fin that is associated with anterior elongation by the presence of outgrowth AER markers *Wnt3, Fgf7*, and 3′Hox genes. O’Shaughnessy [24] demonstrated another novel AER that is associated with development of claspers in the little skate [24]. Claspers are a sexually dimorphic modification to the pelvic fins of male cartilaginous fishes that develop into rolled structures extending from the medial portion of the pelvic fins and are used for sperm transfer during copulation [3, 25]. A recent investigation into the development and outgrowth of claspers examined the genetic pathway driving clasper development [24]. This study found that expression of the AER marker *Fgf8*, in conjunction with *Sonic hedgehog* (*Shh*) pathway (regulated by *Gremlin1* and Androgen Receptor) was sufficient to initiate clasper development. Further, expression of *HoxD12* and *HoxD13* were expressed as sex-specific genes in claspers (i.e., expressed in claspers of males but not in pelvic fins of females). However, the posterior HoxA genes were not investigated, and their role in novel morphologies such as the batoid pectoral fin and male claspers remain undescribed.

Here, we describe novel expression patterns for the posterior HoxA genes, *HoxA11* and *HoxA13,* during the development of modified fin structures in the little skate. Distinct morphologies in the anterior pectoral and pelvic fins appear to be specified by different HoxA genes, and we propose a novel AER in anterior pelvic fin. We also found evidence suggesting that *HoxA11* is associated with the development of the fin rays in the little skate, which contrasts with the role of *HoxA13* in fin ray development in ray-finned fishes [26]. Finally, while *HoxA11* is expressed in a broad domain marking the posterior half of the pelvic fin in males and females, *HoxA13* is expressed exclusively in the posterior claspers of males in the little skate (*Leucoraja erinacea*), a representative batoid, which is somewhat reminiscent with limb patterning in the zeugopod and autopod of tetrapods, but without exclusion of *HoxA11* by *HoxA13.*

## Methods

### Embryos and staging

**Fig. 1:**
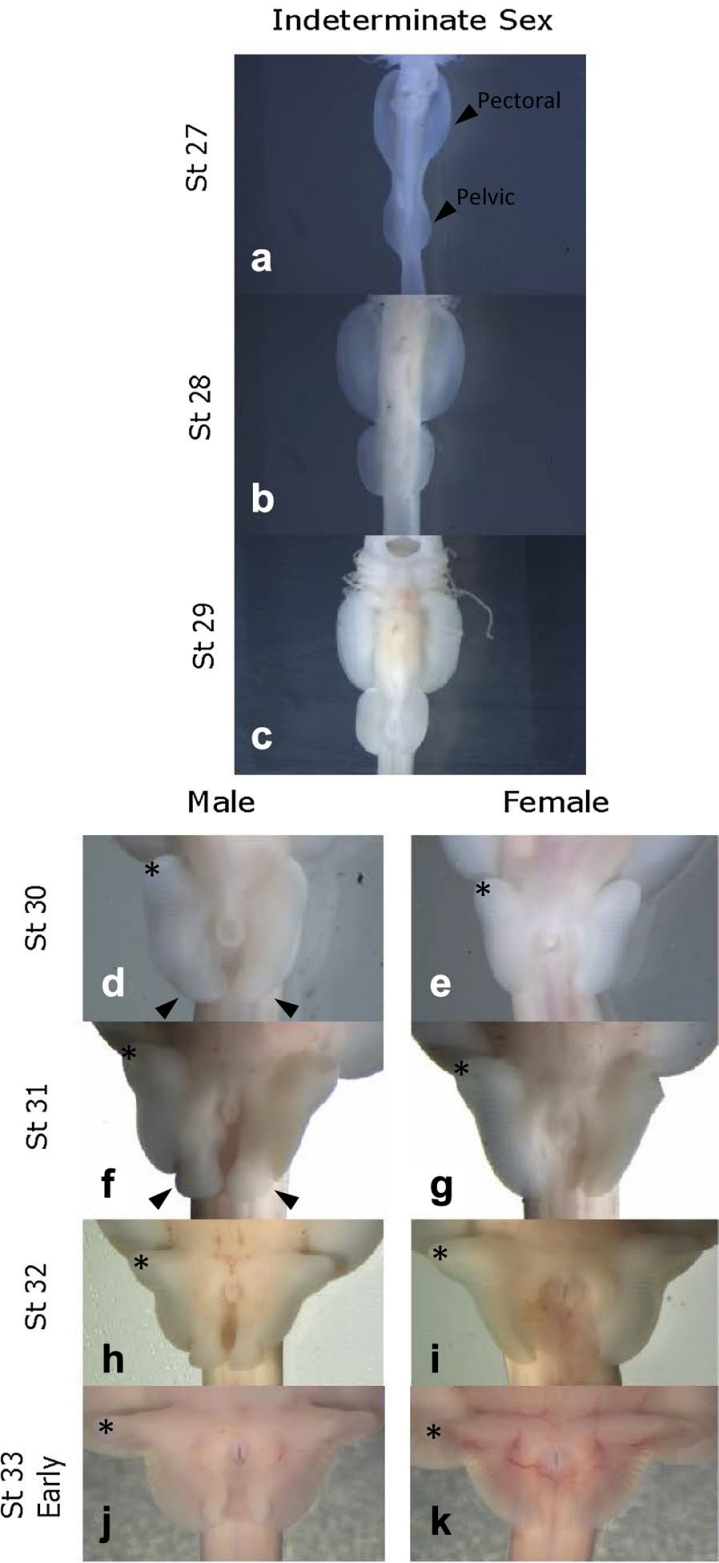
Developmental staging scheme for the little skate, *Leucoraja erinacea*, outlining early pectoral and pelvic fin development (**a**–**c**) and later pelvic fin development (**d**–**k**). Early development shows the paired fin outgrowth from the midline of the body. Sex is indeterminate at these stages (**a**–**c**). At stage 30, clasper morphogenesis begins in males, with visible curvature of the posterior bud (**d**), which is absent in females (**e**). Outgrowth of the crura begins in the anterior pelvic fins at stage 30 as well (**d**, **e**). As development continues the claspers form a separate lobe (**f**) and eventually forms a rolled structure by stage 33 (**j**). Likewise, the crura continue to elongate distally, forming a distinct morphology (**f**–**k**). Asterisks indicate crura on both males and females. Black arrows indicate claspers (**d**, **f**)

Little skate embryos were obtained from the Marine Biological Laboratories (Woods Hole, MA). Embryos were removed from their egg capsules and euthanized by cervical transection. Whole embryos were placed into 4% paraformaldehyde overnight at 4 °C and then transferred to 100% methanol and stored at − 20 °C. Embryos were sexed and staged following the staging scheme of Maxwell et al. [27] for the winter skate (*Leucoraja ocellata*). Gene expression patterns were evaluated at several stages spanning paired fin development, which were stages 27, 28, 29 30, 31, 32 and early 33 (Fig. 1). A total of 205 embryos were used for this study, with 23 of those embryos utilized for clearing and staining and the remainder used for in situ hybridization.

### Clear and stain

Little skate embryos at stages 30, 31, 32 and 33 were cleared and stained following Gillis et al. [3]. In short, embryos in 100% methanol were re-hydrated in gradients of ethanol (100%;70% at 2 h each), then left overnight in 0.2% alcian blue in ethanol containing 30% glacial acetic acid. Embryos were then moved to 100% ethanol with 30% glacial acetic acid for 24 h, followed by 3 days of ethanol gradients (70%;50%;25%) and 24 h of submersion in distilled water. An addition of a 0.5% alizarin red in KOH was added for 24 h submersion, followed by de-staining in 0.5% KOH for up to 24 h, and a graded glycerol series for 3 days in 0.5% KOH before photographing in 80% glycerol.

### Whole-mount in situ hybridization

Probes for in situ hybridization (*ISH*) were synthesized from constructs containing the target insert for HoxA (*HoxA11*, *HoxA13*) and HoxD (*HoxD11*, *HoxD12*, *HoxD13*) genes (Table 1) and cloned using the pGEM-T Vector System II (Promega) and linearized using NcoI, SpeI and SphI (Promega) following Wilkinson [28]. *ISH* was performed following the protocol of Wilkisnson [28], with empirical optimization of bleaching time, hybridization temperature, blocking time and NBT/BCIP staining. Following staining, embryos were re-fixed in 4% paraformaldehyde and photographed in 50% glycerol/50% PBT and stored in 100% glycerol at 4 °C. *Wnt3* expression images were graciously provided by Nakamura et al. [20].

**Table 1:** HoxA and HoxD primers used for in situ hybridization probes in this study

Gene	Forward (5′–3′)	Reverse (5′–3′)
HoxA11	GATGAGCGGGTTCCTTGTGGC	GGTGGAGAAGGAGACGAGTC
HoxA13	GCAGGAATTTGATGGCCCAT	CACCTCTGGAAGTCGAGTCT
HoxD11	GGCCAAGATTTCTCGACAGT	AGTTGACCGAAAAGTCCGTG
HoxD12	CAGCTGGCAAGTCTGTCAC	TCTCCCTCTGTAAATGAAGGC
HoxD13	CTGCATTTGGAGCACATCAC	CTAATGGCTGGAATGGTCAAG

### Sectioning

Following in situ hybridization, embryos for sectioning were placed into 30% sucrose in 1X PBS overnight at 4 °C. Sucrose was removed and embryos were embedded with “O.C.T.” Compound (Tissue Tech), and were immediately frozen on dry ice in plastic molds. Embryos were cryosectioned at 15 µM.

## Results

### *HoxA13* and *HoxA11* specify two novel AER-like domains in distinctly modified regions of the anterior paired fins in the little skate

**Fig. 2:**
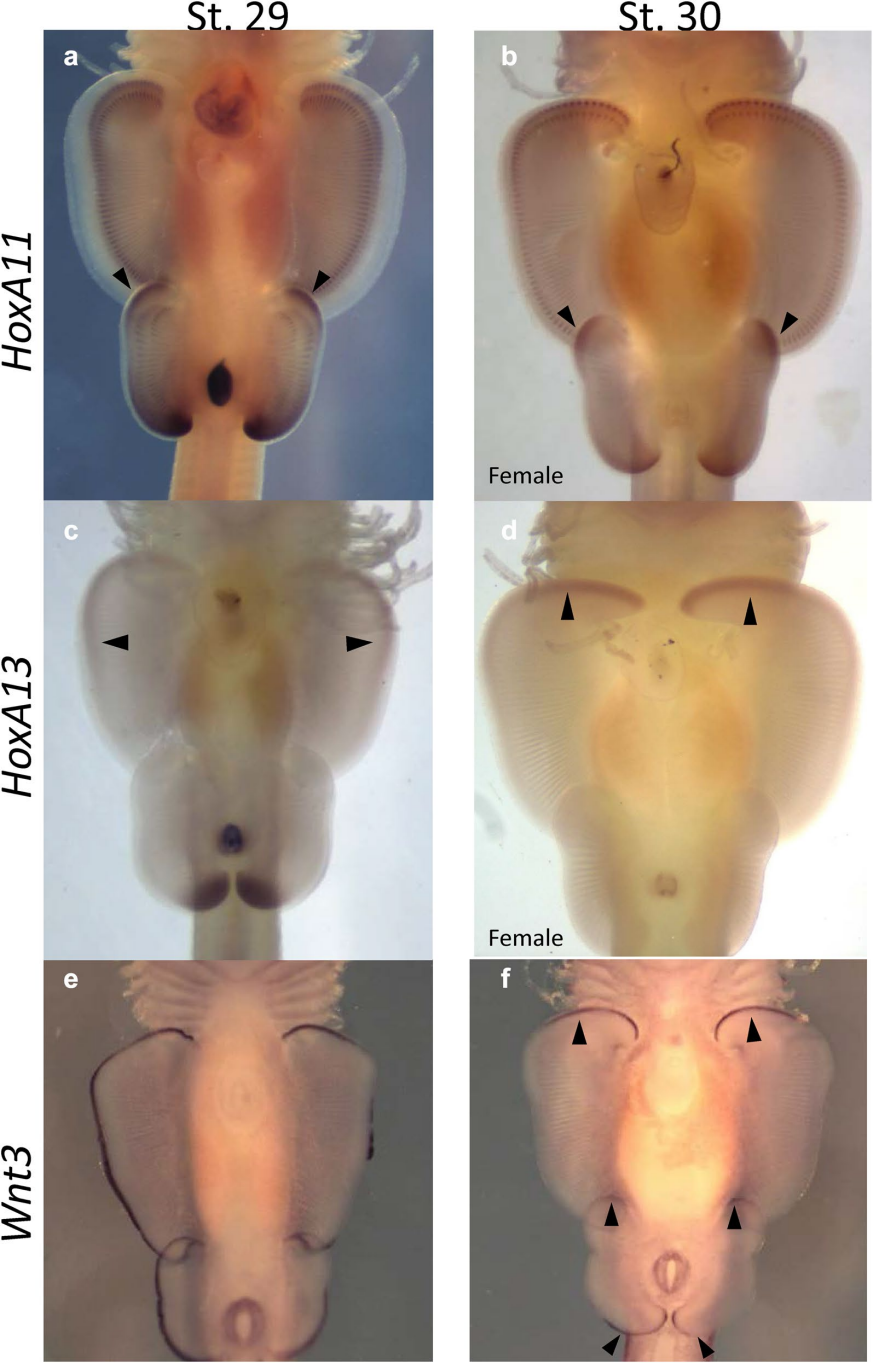
Expression of *HoxA11*, *HoxA13* and *Wnt3* in little skate pectoral and pelvic fins. *HoxA11* expression is located in the anterior pelvic fin, where the crura will elongate (**a**, **b**). In the pectoral fins, *HoxA13* is expressed along the distal portion of the pectoral fin, with expression restricted at the distal mid fin (**c**). By stage 30, *HoxA13* is restricted to the anterior most portion of the pectoral fin (**d**). *Wnt3* is broadly expressed in the entire distal domains of the pectoral and pelvic fin at stage 29 of development, but then recedes to the anterior domains of the pectoral fin and the anterior and posterior domain in the pelvic fin (**e**, **f**). Note that *HoxA11* expression overlaps with *Wnt3* in the anterior pelvic fins **(a**, **b**, **e**, **f**) and *HoxA13* expression in the pectoral fins overlaps with the anterior *Wnt3* expression (**d**, **f**), indicating that *HoxA11* and *HoxA13* are associated with regions of outgrowth and novel AERs. *Wnt3* images are courtesy of Nakamura et al. [20]. Black arrows mark expression

During pectoral fin development in the little skate, *HoxA13* is expressed along a narrow ridge on the distal margin from the anterior tip to the distal mid fin at stage 29 (Fig. 2c). Yet at stage 30, *HoxA13* expression is concentrated in the anterior pectoral fin (Fig. 2d) in a region that has been described as a novel AER based on *Wnt3* expression [20]. While expression of 3′ anterior markers *HoxA2*-*5* in this region [20] is consistent with classic collinear expression, it was somewhat surprising to find expression of the 5′ *HoxA13* gene in the anterior pectoral fin*. Wnt3* exhibits a similar progression with expression along the entire distal margin of the pectoral fin at stage 29, which is then restricted to the anterior pectoral fin at stage 30. By stage 30, *HoxA13* shares an identical expression domain as *Wnt3* in the region of distal outgrowth associated with batoid fin morphogenesis. *HoxA13* is not expressed anywhere else in the developing pectoral fin (Fig. 2); therefore, *HoxA13* expression appears to be exclusively associated with the previously described novel AER domain in little skate pectoral fin.

**Fig. 3:**
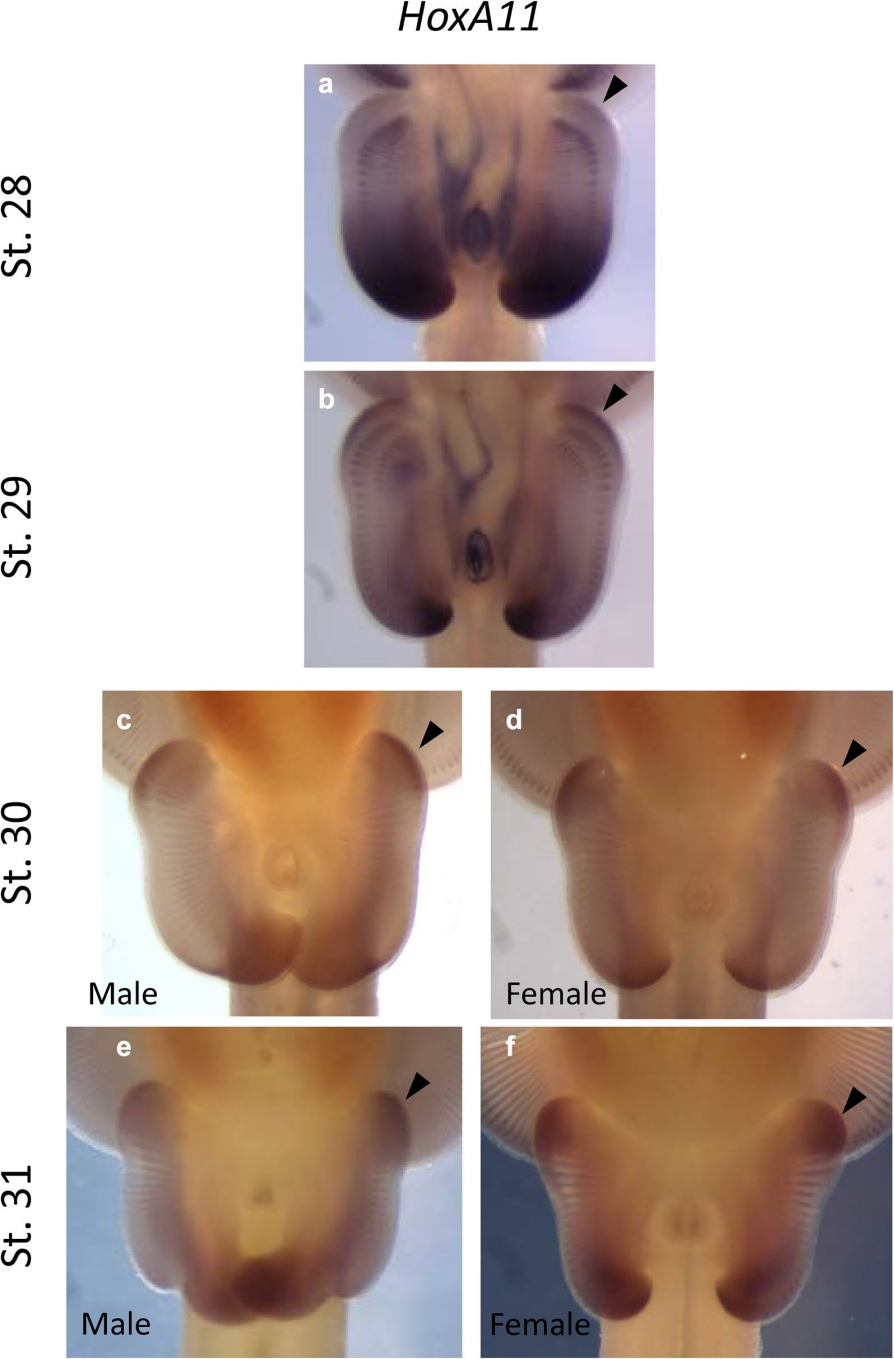
*HoxA11* expression in anterior pelvic fins corresponds to the developing crura. Expression is observed at stage 28 (**a**) and continues through stage 31 (**b**–**f**) in a region that overlaps with *Wnt3* expression (see Fig. 2 F), indicating a novel AER-like domain that is associated with the distal outgrowth of the crura. Interestingly, the crura does not begin to develop until stage 31 (**e**, **f**), indicating that *HoxA11* begins to pattern the crura before elongation occurs. *HoxA11* is expressed in the posterior pelvic fin in both males and females (**c**–**f**)

Interestingly, there is another outgrowth domain in the anterior pelvic fin of the little skate that elongates during morphogenesis, which is distinct from the distal outgrowth that occurs in the anterior pectoral fin. The anterior lobes of the pelvic fin, known as the crura (crus when referring to a singular lobe), are elongated into a “butterfly” shape [29] in the little skate beginning at stage 30 (Fig. 1d, e) [27]. It is likely that this region corresponds to an additional distinct AER based on *Wnt3* expression at stage 30 (Fig. 2f). *Wnt3* is expressed along the entire distal rim of the pelvic fin at stage 29, but by stage 30 was restricted to the anterior pelvic fin corresponding to the crura, and the posterior region of the pelvic fin. In the anterior pelvic fin, *HoxA11* expression overlaps with *Wnt3* expression along a narrow ridge on the distal margin. *HoxA11* expression begins at stage 28, before outgrowth of the crura (Fig. 3a), with peak expression of *HoxA11* in the elongating crura at stage 30 (Fig. 3c, d). *HoxA11* expression continues until developmental stage 31 (Fig. 3e, f), as outgrowth in the crura continues to form a distinctly elongated lobe (Fig. 1f–k).

### The posterior HoxA genes are also expressed in broad domains marking distinct regions during pelvic fin development

**Fig. 4:**
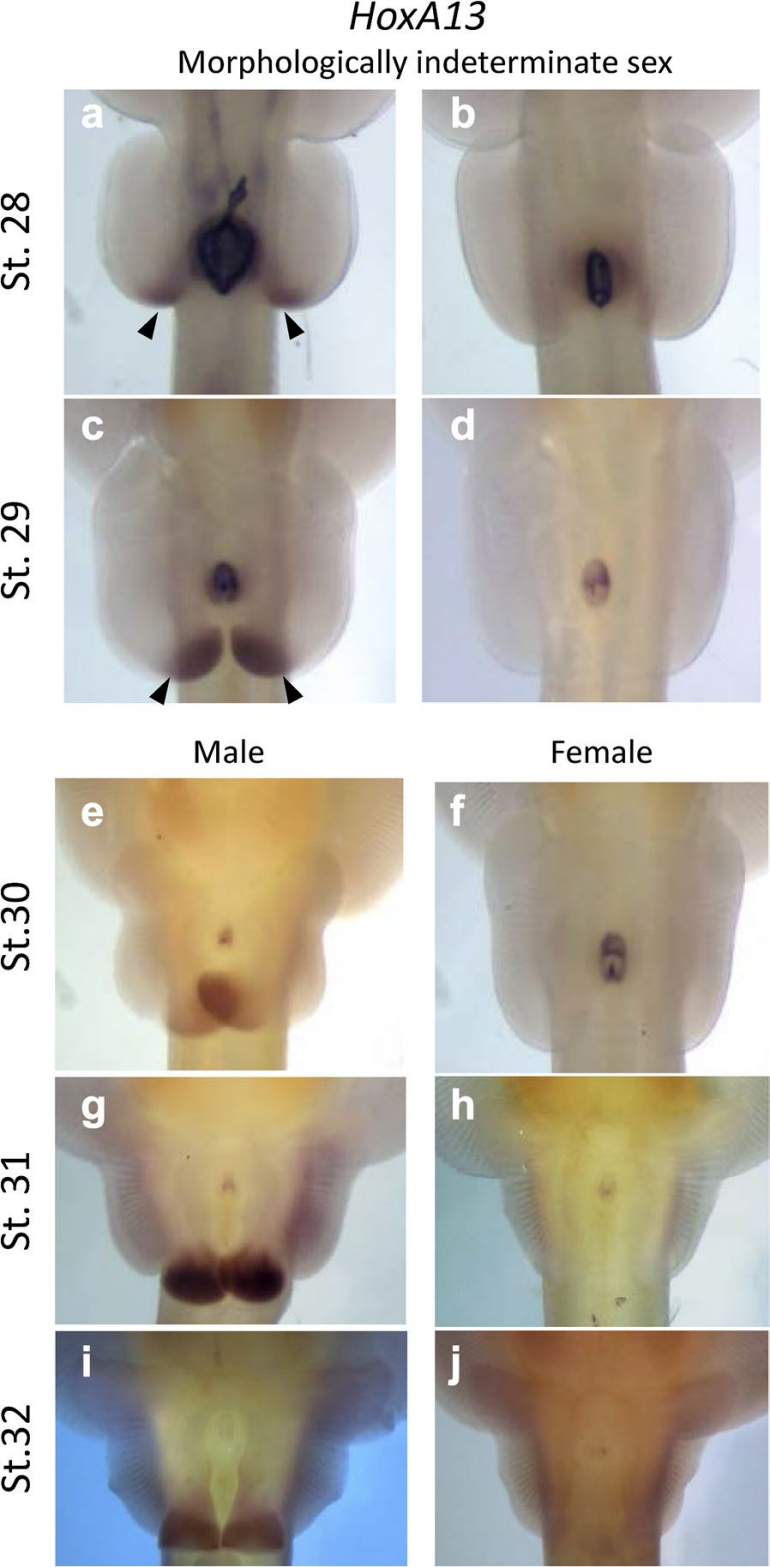
*HoxA13* expression is sexually dimorphic in the claspers of males, and is not expressed in pelvic fins of females. At developmental stages 28 and 29, sex cannot be determined morphologically as the claspers have not yet begun to differentiate, however *HoxA13* expression occurs in half of all embryos (Black arrows **a**, **c**), suggesting that *HoxA13* is patterning the claspers before they form (**a**–**d**). At stages 30–32, clasper morphogenesis is apparent, and *HoxA13* expression continues in males, with no expression in the pelvic fins of females (**e**–**j**)

In pelvic fins of the little skate, *HoxA13* expression is specific to a region corresponding with the early developing claspers (Fig. 1d–k), suggesting a role for specifying this novel domain in ancestral jawed vertebrates. *Wnt3* is expressed along the distal ridge of the posterior pelvic fins, but it is unclear if this is sex specific (Fig. 2e, f). O’Shaughnessy et al. [24] described a novel AER in the developing clasper bud as indicated by sustained *Fgf8*, *Grem1* and *Shh* expression along the posterior outgrowth region of the claspers, that remained present in males well after expression had subsided in posterior pelvic fin in females (which lack claspers). We found *HoxA13* expression occurs exclusively in developing claspers of males (*N* = 15), with no expression in pelvic fins of females (*N* = 10) in the little skate at stages 30–32 (Fig. 4). Further, *HoxA13* is expressed in the region of the clasper anlagen in half of the embryos evaluated at stages 28 and 29 (*n* = 4 st. 28; *n* = 6 st. 29), suggesting sex-specific differences during the onset of clasper specification (Fig. 4a–d), with broad expression continuing in the males until developmental stage 32 (Fig. 4e–j).

*HoxA11* displays another interesting and unique expression pattern in developing pelvic fins, with a broad expression domain that marks the posterior half of the pelvic fin at stage 29 (Fig. 3a). This expression pattern later recedes to the posterior pelvic fin in both males and females by developmental stage 30 (Fig. 3c, d). This broad, posterior expression pattern of *HoxA11* has not been documented previously and may be unique to batoids.

### *HoxA11* expression is associated with fin ray development in paired fins of cartilaginous fishes

**Fig. 5:**
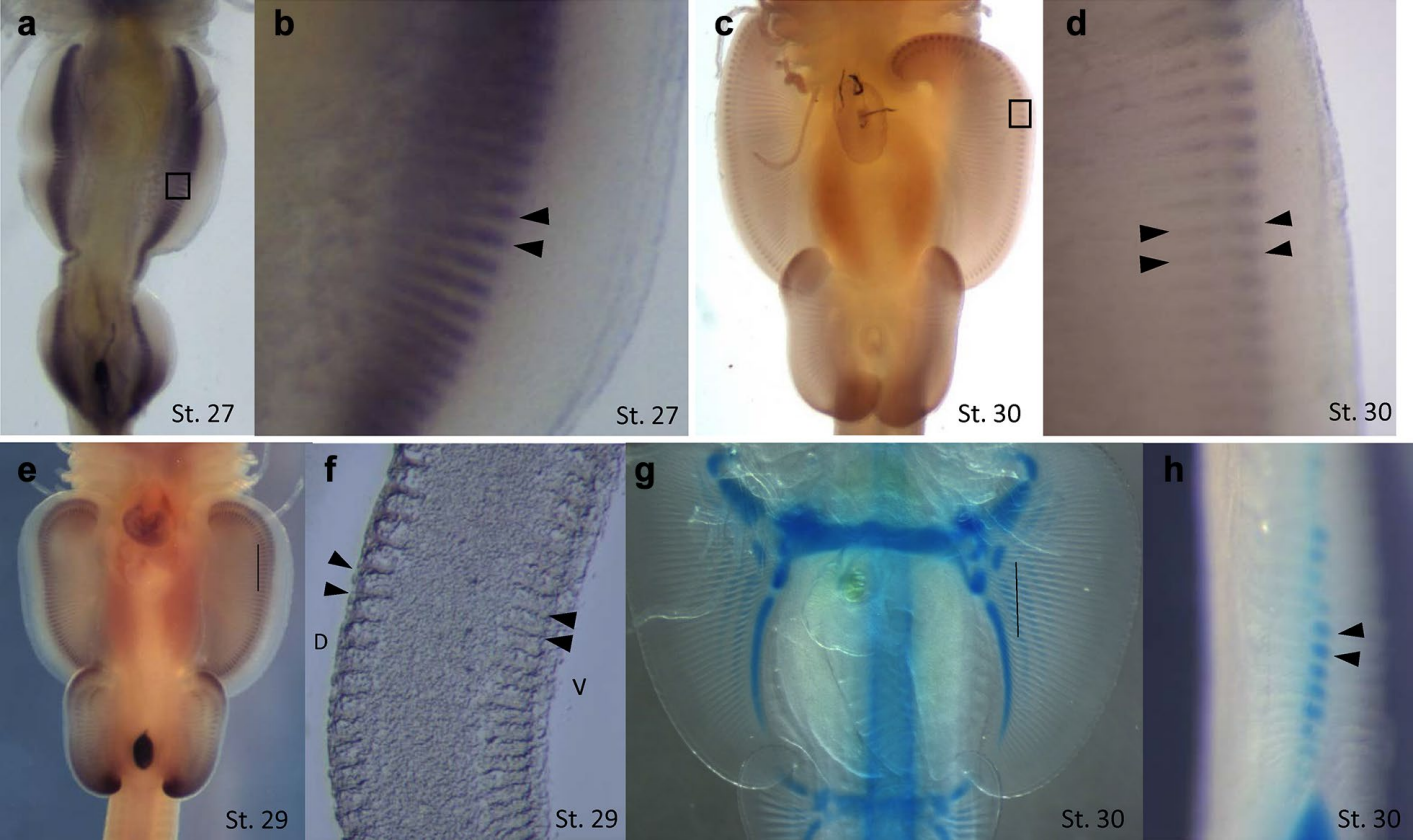
*HoxA11* is associated with the developing fin rays in the pectoral and pelvic fins (**a**–**d**). *HoxA11* expression begins medially in paired fins as a vertical band (**a**) composed of horizontal stripes (**b**) at developmental stage 27. As the fins grow and extends laterally, *HoxA11* expression localizes distally with the fin expansion. Initially, expression is condensed with some stripes of expression merged (**a**), yet by stage 30, expression is associated with individual fin rays (**c**). While it appears that fin ray expression is in two waves, it is actually expressed in two planes, with the more distal expression on the dorsal side of the fin, and the inner expression is on the ventral side of the fin (**d**). Boxes indicate magnified regions. Sectioning the pectoral fin at stage 29 reveals that *HoxA11* expression is not localized in the fin rays per se, rather expression occurs in dorsal and ventral domains marking segments associated with fin ray condensations (**e**, **f**). A lateral section of the pectoral fin in a cleared and stained specimen indicates that the cartilaginous condensation of the fin ray occurs in the mid-mesenchymal tissue of the developing fin (**g**, **h**). Black arrows indicate *HoxA11* expression in the developing fin rays (**b**, **d**, **F**), and cartilaginous condensation (**h**). Black lines indicate where the sections were taken (**e**, **g**). *D* = dorsal, *V* = ventral (**f**)

**Fig. 6:**
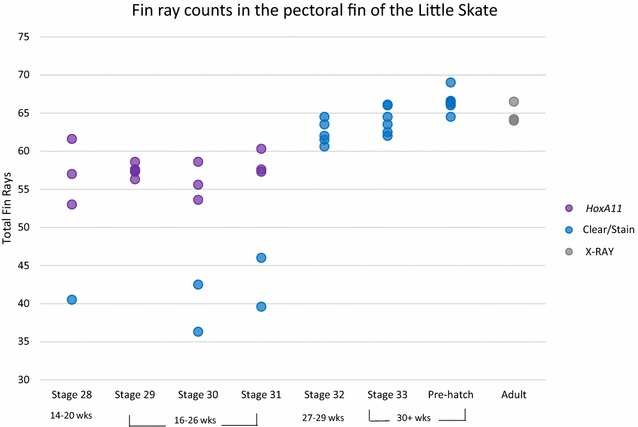
Pectoral fin ray counts of the little skate during development. Blue points indicate number of fin rays in cleared and stained specimens, purple indicates number of *HoxA11* expression stripes in specimens prepared by in situ hybridization, and gray points indicate number of fin rays from radiographs of adult specimens. After 26 weeks of development, expression of *HoxA11* turns off and cartilaginous condensations are present in the developing fin rays, with a total number of 60–69 fin rays per individual. Development in weeks is indicated under developmental stage. *N* = 4, st. 28; *N* = 5, st. 29; *N* = 5, st 30; *N* = 5, st. 31; *N* = 5, st. 32; *N* = 6, st. 33; *N* = 7, Pre-Hatch; *N* = 3, Adult

*HoxA11* marks development of fin rays, composed of cartilaginous radials that begin to develop in the proximal finfold during early outgrowth of paired fins from the body wall (Fig. 5a). As the fins continue to expand distally, *HoxA11* expression follows the distal leading edge of the fin ray elements through developmental stage 31, after which *HoxA11* expression in the fin rays dissipates (see Additional file 2). We compared the number of fin ray elements in embryos of the little skate (by clear and stain specimens), and adults (by radiograph specimens) with bands of *HoxA11* expression in the developing pectoral fins (Fig. 6). We were able to distinguish 53–62 bands of *HoxA11* expression at stages 28–31, which is approximately ten fewer than the total number of elements in mature fins. However, it was difficult to count expression bands precisely on the anterior and posterior fin boundaries where expression bands merged or pelvic fins overlapped; therefore, the number of *HoxA11* bands was likely underestimated (Fig. 6). Nonetheless, it appears that the patterning of fin rays is preceded by *HoxA11* expression before development of cartilaginous condensations, as *HoxA11* is no longer expressed when fin rays mature. For example, at stages 30 and 31 there are over 55–59 bands of *HoxA11* expression, but only 39–43 fin rays in the mid pectoral fin have cartilaginous condensations visible by clear and stain at that stage. Therefore, chondrification of fin rays lags behind *HoxA11* expression and the number of fin rays visible by these two different methods is out of sync. Remarkably, the number of fin ray elements in adults is similar to the number of cartilaginous condensations in pre-hatch embryos, with 60–70 elements clearly visible from stage 32 through adulthood (Fig. 6).

At stages 28 and 29 (Fig. 5e, Additional file 2A, B), *HoxA11* expression is observed in dorsal and ventral planes of the paired fins (i.e., illustrated expression is not offset in a proximal–distal pattern but is actually expressed in overlapping dorsal and ventral planes). We sectioned the pectoral fin of embryos after whole-mount in situ hybridization, which confirmed that *HoxA11* is expressed in two planes (Fig. 5f). However, sectioning of cleared and stained fins at a later stage of development revealed that cartilaginous condensations develop in the middle mesenchymal tissue of the fin, not on the dorsal and ventral planes where *HoxA11* is expressed, suggesting that *HoxA11* specifies regions for fin ray development or marks domains between fin rays, in a dorsal/ventral fashion (Fig. 5f, h).

## Discussion

### Expression of *HoxA13* and *HoxA11* is associated with novel AERs during paired fin morphogenesis in a representative batoid

In the pectoral fin, *HoxA13* is expressed as a narrow stripe along the anterior ridge that corresponds to a novel AER [20] and overlaps with *Wnt3* expression. While the overlapping expression of *HoxA13* and *Wnt3* is indeed intriguing, there are data suggesting a functional role for *HoxA13* in the anterior expansion of the pectoral fin. When little skate embryos are exposed to retinoic acid (RA) treatment, anterior expansion of the pectoral and pelvic fins fails [30], resulting in a more “shark like” morphology. During limb regeneration in Axolotl, the majority of targets directly affected by RA signaling were those in the HoxA cluster, including *HoxA11* and *HoxA13* [31]. RA functions by binding to multiple receptors (RARs/RXRs) before binding to retinoic acid response elements (RAREs) in the regulatory regions of target genes [32]. Not surprisingly, a RARE has been identified upstream of the HoxA cluster in zebrafish and mice [33].

In addition to demonstrating that *HoxA13* expression is associated with a novel AER in the anterior pectoral fin (Fig. 2c, d), we also provide evidence that *HoxA11* expression is associated with a morphological adaptation in anterior pelvic fin (Fig. 2a, b). Skates are adapted to benthic lifestyles, with elongated anterior pelvic fin lobes, called crura, that are utilized for an alternative mode of locomotion known as “punting”, which is thought to be a more energy efficient mode of locomotion along the sea floor [29, 34]. We propose that the region corresponding to the crura is specified by an additional novel AER-like domain based on *Wnt3* expression that was identified but not highlighted in Nakamura et al. [20]. Fin expansion and elongation in both the anterior pectoral and pelvic fins failed when exposed to the WNT inhibitor IWR1 [20] supporting the role of AER outgrowth via *Wnt3* expression in the morphological evolution of both distinct anterior fin domains in the little skate. While O’Shaughnessy et al. [24] described expression of several genes in the *Shh* pathway that are associated with clasper development in the posterior pelvic fins of males, they did not discuss expression in the anterior pelvic fin associated with the outgrowth of the crura. However, a few of these genes, *Grem1*, *Ptch1*, and *Shh* appear to be expressed in this anterior domain. One possibility is that RA treatment could interrupt the *Shh* pathway, and therefore affect HoxA expression indirectly. However, *Shh* is not expressed in anterior pectoral fin in the little skate at stages 29–30, ([20] and pers. communication Jeff Klomp), and *Grem1* expression is biased toward the posterior pectoral fin [20]. Therefore, failure of anterior development in both pectoral and pelvic fins after exposure to RA [30, 35] suggests a more direct link between RA and HoxA, and is consistent with a functional role for *HoxA13* and *HoxA11* in specifying novel morphologies in anterior paired fins. These observations are consistent with the idea that derived lineages that have undergone fin and limb modifications exhibit novel expression patterns of *HoxA11* and *HoxA13* that are associated with development of unique features, such as the proximo-distal expression pattern in the zeugopod and autopod of tetrapods, and the extended anterior paired fins of batoids as selective advantages for different habitats and life histories. While there appear to be many novel AERs associated with these outgrowths and modifications, each is associated with a unique HoxA code that may be associated with domain specification and identity.

### *HoxA11* and *HoxA13* mark broad domains in posterior regions of fin/limbs with novel morphologies

*HoxA13* expression occurs in a broad domain that is exclusive to the developing claspers. Claspers begin to bud from the posterior pelvic fin at developmental stage 30, meaning that earlier stages of the little skate cannot be sexed. Yet differences were noted in *HoxA13* expression prior to clasper morphogenesis, with half the embryos displaying *HoxA13* expression at stages 28 and 29 (Fig. 4a–d), suggesting that *HoxA13* sets up early patterning of the claspers. The posterior HoxD genes, *HoxD12* and *HoxD13,* are also sexually dimorphic, with no expression in females, and expression domains restricted to the developing claspers (see Additional file 1A–H) [24]. There was no *HoxA13* expression in the posterior regions of developing pectoral or pelvic fins in the little skate, contrary to what has been observed in the fins of catshark [10], zebrafish [13] and paddlefish [11], and the limbs of frog [7] and chick [8], suggesting that altering the Hox code in specific lineages is associated with specific novel morphologies.

*HoxA11* is expressed in both males and females during pelvic fin development (Fig. 3c–f), with expression in the anterior region of the crura, and the posterior half of the pelvic fin, including the claspers of males. The broad expression domain of *HoxA11* in posterior pelvic fins may be unique to batoids. However, *HoxA11* also has a broad expression domain in the paddlefish pectoral fin bud [11], and  in the forearm (zeugopod) in tetrapods that is excluded in the distal autopod by *HoxA13* [9, 36, 37]. It is worth noting that tetrapod forelimbs are homologous with the posterior region of paired fins (metapterygium or basipterygium) in cartilaginous fishes. Regardless, we observe a broad expression domain of *HoxA11* in the posterior pelvic fin/clasper, while *HoxA13* exhibits a broad expression domain that is specific to the developing claspers. These data suggest that the specific expression patterns of *HoxA11* and *HoxA13* are associated with the evolution and development of novel fin/limb morphologies in the zeugopod, autopod and claspers, and the role of *HoxA11* in posterior pelvic fin warrants further investigation.

### *HoxA11* is associated with development of support structures in pectoral and pelvic fins

*HoxA11* is expressed in association with the developing fin rays in pectoral and pelvic fins of the little skate. This expression pattern was also illustrated, but not described, in the developing fins of the catshark [10, 38]. In zebrafish, *HoxA13* expression is associated with the developing fin rays [26], but *HoxA11* was not evaluated. We found no evidence for *HoxA13* expression in the fin rays in the little skate, despite strong *HoxA13* expression in other areas of fin outgrowth and development. This suggests that *HoxA11* is associated with patterning of fin rays in the cartilaginous fishes, and *HoxA13* may have been coopted in fin ray expression in ray-finned fishes.

By comparing *HoxA11* expression bands in pectoral fins with cartilaginous condensations in cleared and stained juveniles and X-rays of older specimens, we discovered that the number of fin rays is specified early in development (Fig. 6), which is preceded by stripes of *HoxA11* expression along the dorsal and ventral margins of paired fins, followed by chondrification in the mid-mesenchymal tissue (Fig. 5e–h). We can observe what appears to be waves of cartilage that may correspond to the individual radial elements of the fin rays at stage 30 (Fig. 5g).

Interestingly, paired fins develop from continuous finfolds that extend laterally down the sides of the body in the little skate, from the region posterior to the head to the cloaca (Fig. 1). At stage 27, *HoxA11* expression occurs in a continuous field along this entire domain (Fig. 5a) marking early patterning of fin rays. Yet by stage 28, the pectoral and pelvic fins are differentiated, and *HoxA11* expression and the finfold tissue between them is no longer present (see Additional file 2B). This developmental scheme is consistent with the finfold theory proposed by Thacher [39], which suggests that paired fins evolved as the “retained portions of a continuous lateral fin” on either side of the body (Mivart [40] and Balfour [41], and described in Tulenko, McCauley [42]). Freitas [43] argued that paired fins evolved from median fins based on shared HoxD expression patterns. HoxA expression patterns also inform aspects of the origin of fin/limbs such as the *HoxA11* pattern we see in fin rays of the little skate, but to our knowledge HoxA expression has not been evaluated in medial fins of jawless or ancestral jawed vertebrates.

## Conclusions

**Fig. 7:**
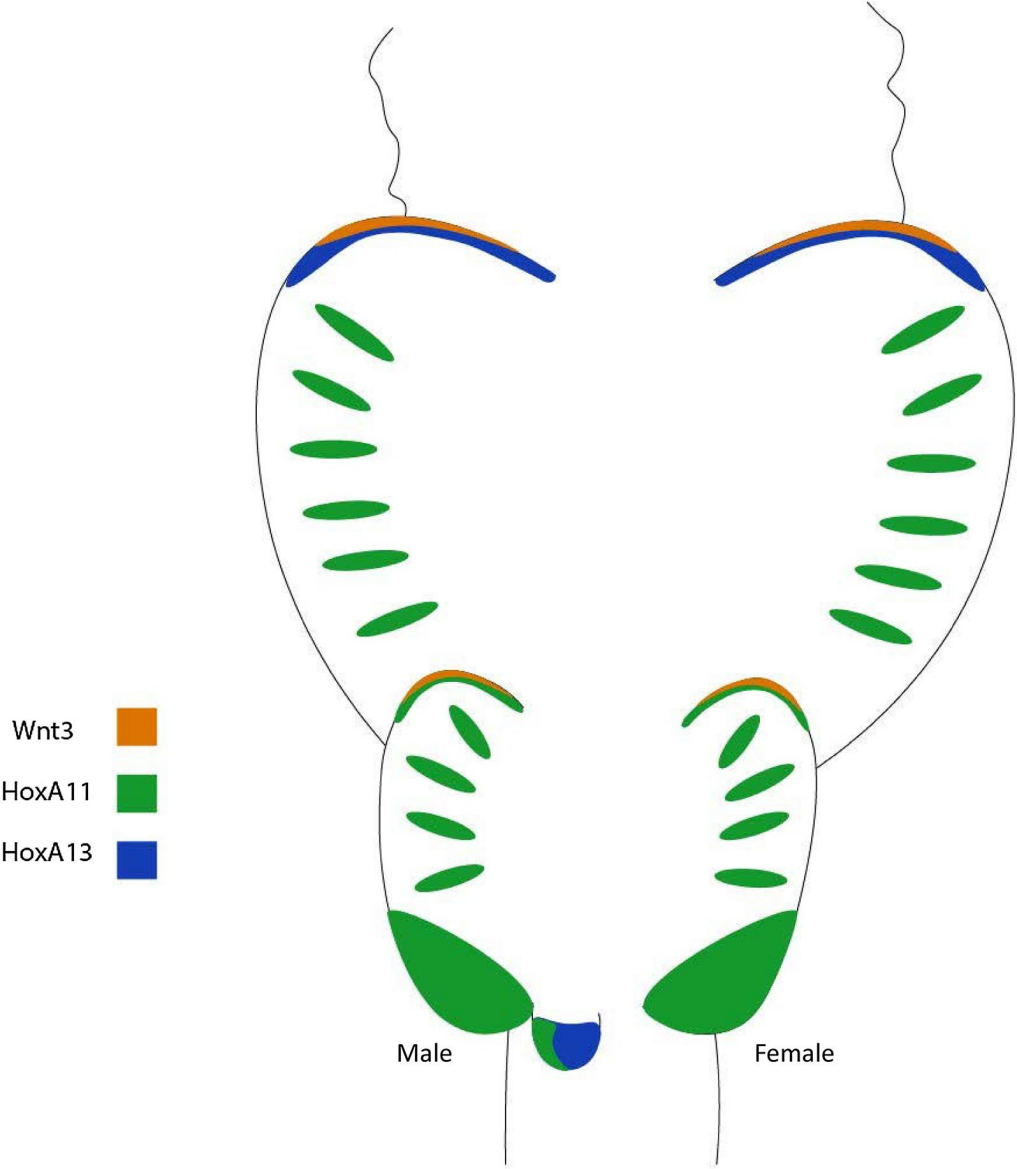
Cartoon illustrating expression of *HoxA11* and *HoxA13* during development of paired fins in the little skate (*Leucoraja erinacea*). Expression of *HoxA13* and *HoxA11* mirror expression of *Wnt3* in novel AER domains in the anterior pectoral and pelvic fins, respectively. *Wnt3* is expressed in both anterior AER domains, but each is patterned by a different HoxA gene. *HoxA11* expression is associated with developing fin rays, and marking a broad domain in the posterior half of pelvic fin at stage 28, which attenuates to posterior pelvic fin by stage 29. While *HoxA11* is expressed in the pelvic fins of both males and females, at stage 32, *HoxA11* is in clasper only, and we see a reverse collinear expression pattern, where *HoxA13* is broadly expressed in the entire clasper while *HoxA11* is a more restricted domain (see Additional file 3). *Wnt3* expression provided by Nakamura et al. [20], while photographs of pelvic fin expression provided by Tetsuya Nakamura

The batoids exhibit fin modifications that provide a unique opportunity to investigate the role of Hox genes in novel morphologies. HoxA genes are relatively understudied and we provide evidence that the posterior HoxA genes play distinct roles in two novel AERs during skate fin development, one of which is newly described here. Further, the little skate exemplifies unique Hox expression patterns differing from the catshark, including large posterior expression domains of *HoxA11* in the pelvic fin (Fig. 7). *HoxA11* is also associated with the development and patterning of fin rays in cartilaginous fishes, in contrast to *HoxA13* patterning fin rays in ray-finned fishes. Here, we have shown that the posterior HoxA genes appear to play a significant role in paired fin morphogenesis in batoids, and it is intriguing that each of the novel fin domains described is associated with a discrete HoxA code that is consistent with their unique specification.

## Additional files

**Additional file 1.** HoxD expression in the pelvic fin of the little skate during early to late development (**A**–**H**). Note that both *HoxD12* (**A**–**B**) and *HoxD13* (**C**–**H**) are expressed exclusively in the claspers and show no expression in the female pelvic fin. *HoxD12* is expressed in the posterior pectoral fin (I), *HoxD13* shows no expression at stage 30 in the pectoral fin (**J**). Similarly, the posterior HoxA genes are not expressed in the posterior pectoral fin, indicating a unique Hox code that specifies specific morphologies during development.

**Additional file 2.** *HoxA11* is associated with the developing fin rays in the pectoral and pelvic fins (**A**–**E**). *HoxA11* expression begins medially in the pectoral and pelvic fins in small stripes (**A**). As the fins continue to elongate, *HoxA11* expression elongates distally, until stage 31 which is the last-stage expression is noted (**E**).

**Additional file 3.** Reverse collinear (RC) expression in the claspers of the little skate. RC expression occurs when the more posterior gene has a broader expression range than the gene anterior to it. In this case, *HoxA13* is expressed throughout the entire clasper (**B**), whereas *HoxA11* is restricted to the distal region (**A**). This expression pattern is likely setting up left–right asymmetry in the claspers and is only observed at stage 32 of development. Arrows denote *HoxA11* expression in the distal claspers.
