# Supplementary material for: The role of HoxA11 and HoxA13 in the evolution of novel fin morphologies in a representative batoid (Leucoraja erinacea)
Source: EvoDevo. 2017 Dec 1;8:24. doi: 10.1186/s13227-017-0088-4 (PMC5709974; doi:10.1186/s13227-017-0088-4)
Supplement: Supplementary file 1 — Additional file 1. HoxD expression in the pelvic fin of the little skate during early to late development (A–H). Note that both HoxD12 (A–B) and HoxD13 (C–H) are expressed exclusively in the claspers and show no expression in the female pelvic fin. HoxD12 is expressed in the posterior pectoral fin (I), HoxD13 shows no expression at stage 30 in the pectoral fin (J). Similarly, the posterior HoxA genes are not expressed in the posterior pectoral fin, indicating a unique Hox code that specifies specific morphologies during development. [file 13227_2017_88_MOESM1_ESM.pptx]

## Slide 1
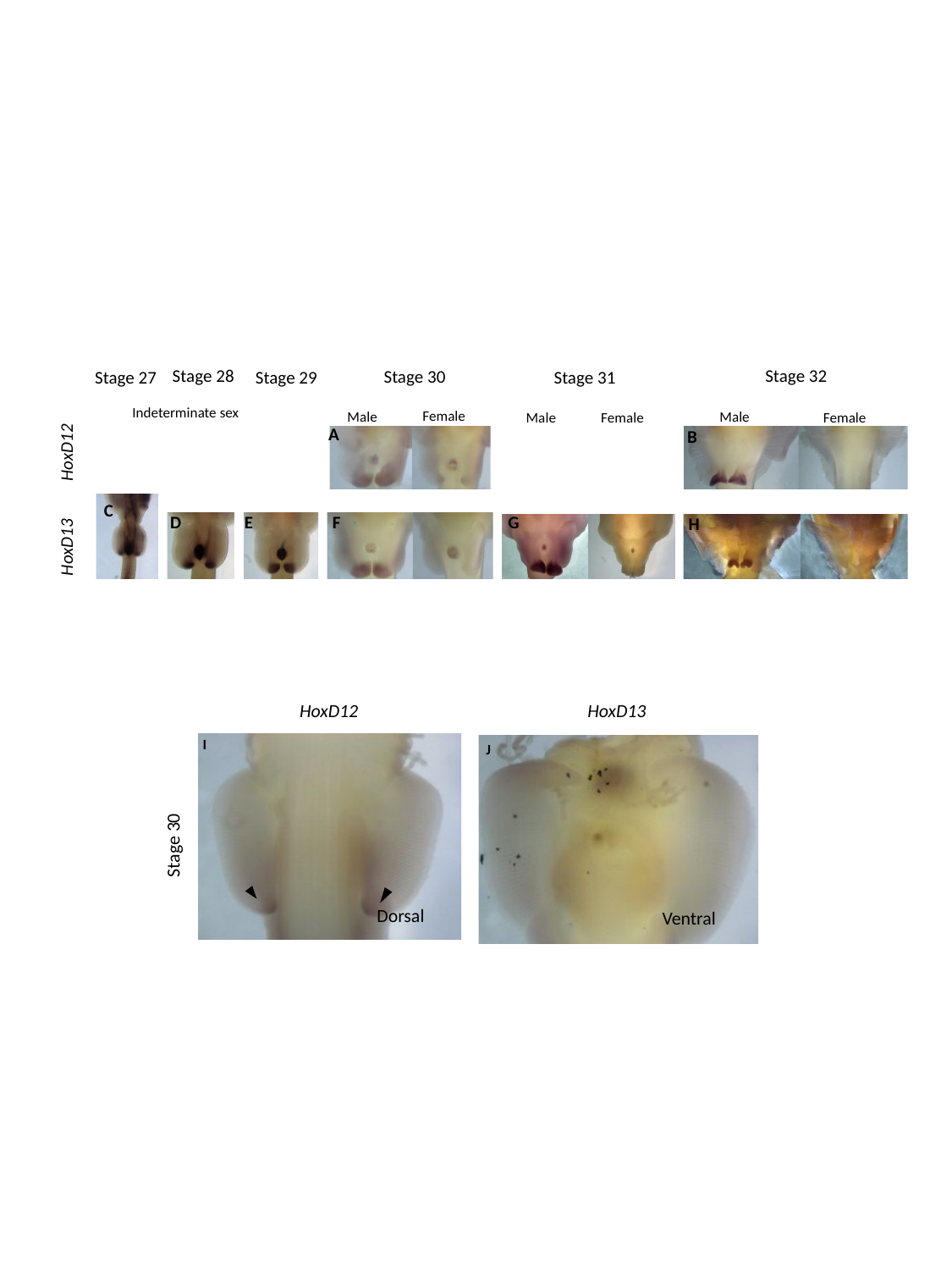

Stage 32
Stage 28
Stage 30
Stage 31
Stage 27
Stage 29
Indeterminate sex
Female
Male
Male
Female
Male
Female
A
B
HoxD12
C
G
D
E
F
H
HoxD13
HoxD12
HoxD13
I
Dorsal
J
Ventral
Stage 30
