# Supplementary figures and images for: The role of HoxA11 and HoxA13 in the evolution of novel fin morphologies in a representative batoid (Leucoraja erinacea)
Source: EvoDevo. 2017 Dec 1;8:24. doi: 10.1186/s13227-017-0088-4 (PMC5709974; doi:10.1186/s13227-017-0088-4)

## Slide 1
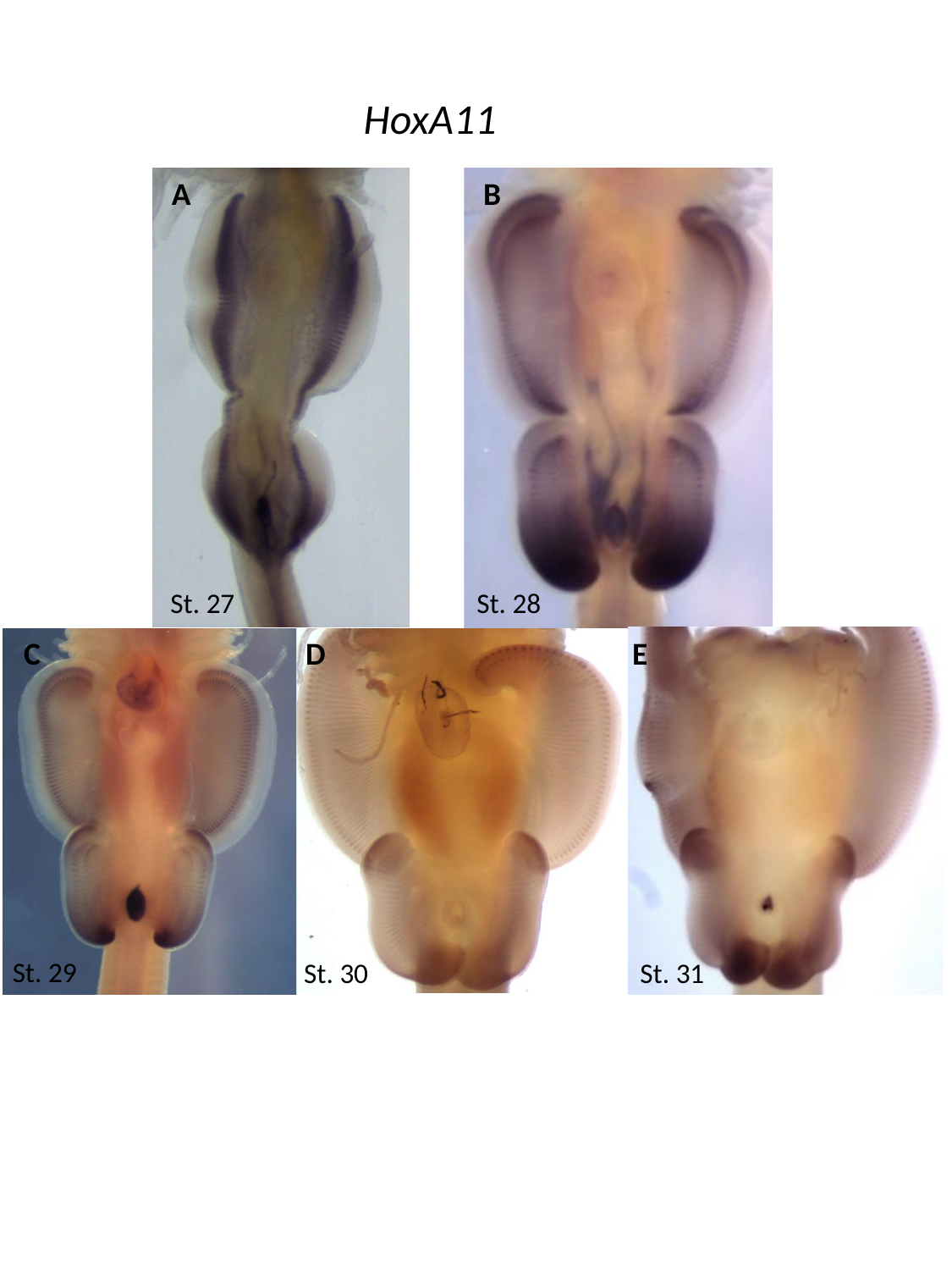

HoxA11
A
B
St. 27
St. 28
C
D
E
St. 29
St. 30
St. 31

Supplement: Supplementary file 2 — Additional file 2. HoxA11 is associated with the developing fin rays in the pectoral and pelvic fins (A–E). HoxA11 expression begins medially in the pectoral and pelvic fins in small stripes (A). As the fins continue to elongate, HoxA11 expression elongates distally, until stage 31 which is the last-stage expression is noted (E). [file 13227_2017_88_MOESM2_ESM.pptx]

## Slide 1
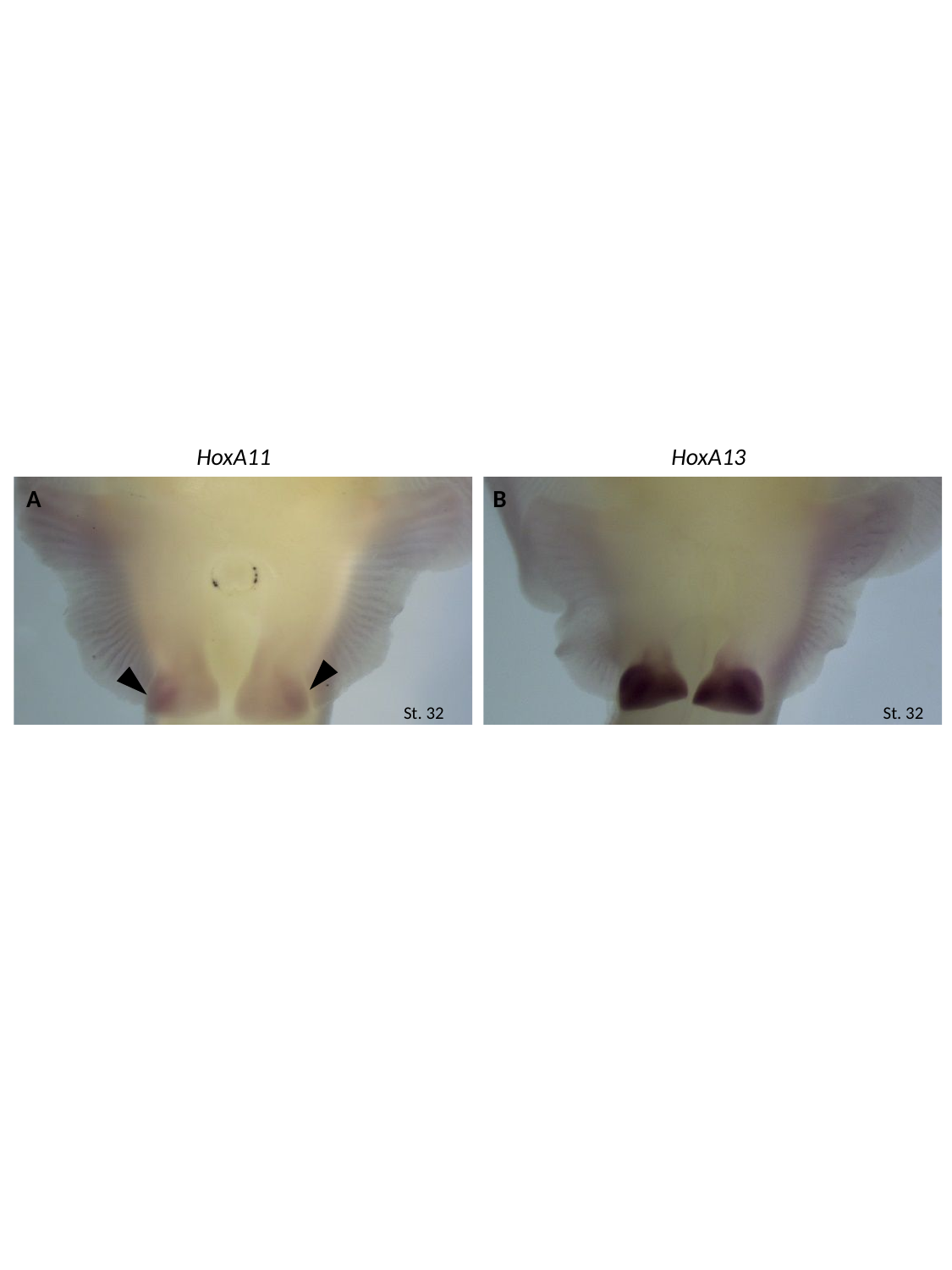

HoxA11
HoxA13
B
A
St. 32
St. 32

Supplement: Supplementary file 3 — Additional file 3. Reverse collinear (RC) expression in the claspers of the little skate. RC expression occurs when the more posterior gene has a broader expression range than the gene anterior to it. In this case, HoxA13 is expressed throughout the entire clasper (B), whereas HoxA11 is restricted to the distal region (A). This expression pattern is likely setting up left–right asymmetry in the claspers and is only observed at stage 32 of development. Arrows denote HoxA11 expression in the distal claspers. [file 13227_2017_88_MOESM3_ESM.pptx]
